# Contagiousness of Cholera

**Published:** 1867-08

**Authors:** 


### *Contagiousness of Cholera.*

None of our readers should fail to read the highly suggestive and valuable article by our excellent colleague, Prof. Rea, furnished in the present number of THE JOURNAL.

